# Environmental assessment of physical-chemical features of Lake Nasser, Egypt

**DOI:** 10.1007/s11356-020-08366-3

**Published:** 2020-04-01

**Authors:** Roquia Rizk, Tatjána Juzsakova, Igor Cretescu, Mohamed Rawash, Viktor Sebestyén, Cuong Le Phuoc, Zsófia Kovács, Endre Domokos, Ákos Rédey, Hesham Shafik

**Affiliations:** 1 Institute of Environmental Engineering, University of Pannonia, Veszprém, 8200 Hungary; 2 Faculty of Chemical Engineering and Environmental Protection, Gheorghe Asachi Technical University of Iasi, Iasi, Romania; 3 Department of Animal Science, Georgikon Faculty, University of Pannonia, Keszthely, 8630 Hungary; 4 Da Nang University of Science and Technology, The University of Da Nang, Da Nang, Vietnam; 5 Department of Botany, Faculty of Science, Port Said University, Port Said, Egypt

**Keywords:** Water quality assessment, Physico-chemical parameters, Aquatic environmental index, Lake Nasser, River Nile, Egypt

## Abstract

Lake Nasser is one of the largest man-made lakes on earth. It has a vital importance to Egypt for several decades because of the safe water supply of the country. Therefore, the water quality of the Lake Nasser must be profoundly investigated, and physico-chemical parameter changes of the water of the Lake Nasser should be continuously monitored and assessed. This work describes the present state of the physico-chemical (nitrate-nitrogen, nitrite-nitrogen, orthophosphate, total phosphate content, dissolved oxygen content, chemical oxygen demand, and biological oxygen demand) water parameters of Lake Nasser in Egypt at nine measurement sites along the Lake Nasser. The algorithm was devised at the University of Pannonia, Hungary, for the evaluation of the water quality. The aquatic environmental indices determined alongside the Lake Nasser fall into the category of “good” water quality at seven sampling sites and exhibited “excellent” water quality at two sampling sites according to Egyptian Governmental Decree No. 92/2013. In light of the tremendous demand for safe and healthy water supply in Egypt and international requirements, the water quality assessment is a very important tool for providing reliable information on the water quality. The protocol for water quality assessment could significantly contribute to the provision of high-quality water supply in Egypt. In conclusion, it can be stated that the parameters under investigation in different regions of Lake Nasser fall within the permissible ranges and the water of the Lake has good quality for drinking, irrigation, and fish cultures according to Egyptian standards; however, according to European specifications, there are steps to be accomplished for future water quality improvement.

## Introduction

The River Nile is the most important freshwater supply in Egypt for thousands of years; it provides renewable water supply such as drinking, irrigation, and canalization in the Nile Valley and the Delta Region (Goher et al. 2014; Ghodeif et al. 2016; Negm 2017). Lake Nasser was generated by the construction of the Aswan High Dam between January 1964 and June 1968 (Abd El-Monsef et al. 2015; El Gamal and Zaki 2017; Salih et al. 2019). The area of the Lake is about 5000 km^2^ (Farhat and Aly 2018). The Lake has a high water storage capacity of 150–165 km^3^ providing a maximum water flow of 11,000 m^3^/s. The mean depth of Lake Nasser is 90 m (Abou El Ella and El Samman 2010; El Shemy 2010), and the maximum width of the Lake is about 60 km (Khalifa et al. 2015). Eastern Desert is the east border of the lake, which contains many precious mineral resources including metals and is bordered on the west by the Western Desert, which contains deposits, limestone, agricultural lands, and archeological sites (Yousif 2019; Hamimi et al. 2020). Due to the regional climate conditions prevailing in Upper Egypt, chlorophyll-a content is considered to be the best descriptive parameter to indicate phytoplankton biomass in freshwater lakes (Liu et al. 2019). The chlorophyll-a content can be positively correlated with temperature, which affects the phytoplankton growth. The temperature represents one of the main abiotic factors responsible for controlling several freshwater physical-chemistry parameters (Hecky 2000; Mohamed 2000). The lowest dissolved oxygen concentrations represent the proliferation of biological organisms (Gupta and Gupta 2006; Reygondeau et al. 2017), which consume the dissolved oxygen content of the water. The dissolved oxygen content is strongly affected by high temperature which is usually higher from November to April and is low from May to October (Yang et al. 2018). The increase in water temperature decreases the solubility of oxygen in water (El-Shabrawy 1996; Toufeek and Korium 2009; Idowu et al. 2013). It is mainly affected by the climatic changes typical for East Africa (El Gammal 2010). Given the obligation of the countries of the world, it is necessary to take preventive measures to slow down and reverse the harmful global environmental processes. Joint actions have been initiated to deal with global and regional environmental issues (Hefny and Amer 2005; Soulie 2013).

Environmental impact assessment (EIA) is an effective tool for conducting an assessment of the environmental impacts of various actions, projects, and investments (Utasi et al. 2013). It is the systematic evaluation of the impacts of human activities on the environment (Rédey et al. 2002). The environmental impact assessment supports the procedures to improve the environmental quality and provides technical support to prevent and eliminate the future environmental damages (Toro et al. 2013).

In light of the international requirements defined by Water Framework Directive and the huge Egyptian demand for pure and potable water supply, the water quality assessment is a very important tool to follow the variations in water quality of Lake Nasser. The aquatic environmental assessment method elaborated by Németh et al. (2017), the water quality assessment technique, and the outcome of the assessment could significantly contribute to the provision of high-quality water from the Lake Nasser and to monitor the water supply lines in Egypt. Therefore, the aim of the work was to study and determine the water quality of the Lake Nasser by applying the aquatic environmental assessment (AEA) method according to Egyptian Governmental Decree No. 92/2013 (GD 2013) in order to make recommendations for water quality improvement on the basis of conclusions of the study.

## Experimental

### Measurements and methods

**Fig. 1:**
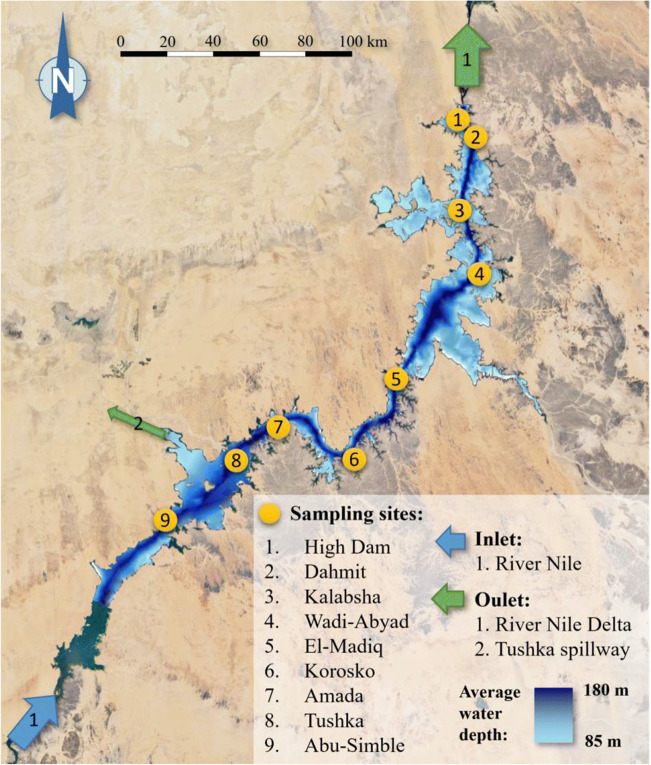
Sampling sites and main water supplies/inlets and discharge/outlet points of the Lake Nasser

The measurements within the present study were conducted at nine measuring points alongside the Lake Nasser which are depicted in Fig. 1. Fourteen water chemistry parameters were measured as given in Table 1. It is to be emphasized repeatedly that the present paper focuses only on the physical-chemistry water parameters, hereinafter referred to as water chemistry parameters. The measurements were carried out in April–May, 2018, and the water samples were taken from the 1-m depth along the main channel of Lake Nasser. The location of the measuring points, inlet, and outlet points is indicated in Fig. 1. The names of the sampling sites are also given in Fig. 1.

**Table 1:** Measured water chemistry parameters around Nasser Lake and their limit values according to Egyptian Governmental Decree No. 92/2013

Parameter	Measuring points/sites									
	1 High Dam	2 Dahmit	3 Kalabsha	4 Wadi-Abyad	5 El-Madiq	6 Korosko	7 Amada	8 Tushka	9 Abu-Simble	Limit value (Governmental Decree No. 92/2013)
Chl-a (μg/L)	11.00	10.80	11.30	13.00	9.85	10.00	9.00	11.90	9.80	16.60
pH	7.66	7.97	8.32	7.84	7.80	7.90	7.81	7.83	7.87	10.00
NTU	8.00	7.80	10.00	10.80	6.00	5.60	6.00	5.00	5.00	25.00
EC (μs/cm)	250	250	250	245	240	232	230	230	235	1000
DO (mg/L)	4.50	5.20	5.00	4.50	5.40	5.20	4.90	6.30	6.70	6.66
COD (mg/L)	8.70	7.80	8.55	11.00	7.60	7.60	8.00	6.00	6.50	10.00
BOD_5_ (mg/L)	1.37	1.20	1.40	1.90	0.90	0.90	1.80	1.50	1.00	6.00
NH_4_ (μg/L)	10.00	7.00	8.00	10.00	6.00	7.00	10.00	5.00	5.00	330
NO_3_ (μg /L)	2000	2000	2200	2000	1900	1900	1900	2100	1600	2000
NO_2_ (μg/L)	12.00	10.00	10.00	10.00	13.00	12.00	13.00	5.00	5.00	20.00
PO_4_ (μg/L)	220	170	160	280	100	70.0	200	220	90.0	500
TP (μg/L)	400	390	430	470	340	350	420	430	291	2000
TSS (mg/L)	17	17	18	18	16	16	17	18	16	50
Fecal coliform (no./100 ml)	30	25	30	28	15	25	28	40	15	200

The aquatic environmental assessment method is illustrated in details for measurement site no. 1 commonly known as Aswan High Dam, and the assessments were carried out according to Egyptian Governmental Decree No. 92/2013 (GD 2013). Data summarized in Table 1 show the measured water chemistry parameters and the measurement results for the Lake Nasser on the basis of analyses carried out at nine measurement sites. The investigations covered the water parameters stipulated in the WFD 2000/60/EC (WFD 2000). Water parameters were measured according to the stipulations of the American Public Health Association (APHA 1985, 2005; NEERI 1991). The water samples of Lake Nasser were taken by using a 1.5-dm^3^ Ruttner sampler and were analyzed for the following water chemistry parameters at site: turbidity (NTU), pH measurements, electrical conductivity, and dissolved oxygen content. The pH measuring device was a glass electrode pH meter (Orion model 601/digital ion analyzer); the electrical conductivity (EC) was measured by using Amber Science Inc., San Diego, conductivity meter, model 1062; and dissolved oxygen content (DO) was determined by Winkler titration method (APHA 2005). Water samples (3 L from each site) were taken and transferred directly into an ice box, and a predefined volume of water was filtered through a glass microfiber filter (GF/F, 0.45-μm membrane), and the samples were refrigerated till further analyses. The following water chemistry parameters were measured at the laboratory of Cairo University according to APHA standard method (APHA 1985; APHA 2005 and NEERI 1991):

- BOD_5_ was determined as per standard method (NEERI 1991);
- COD was determined by potassium dichromate open reflex method (NEERI 1991);
- Ammonium-nitrogen content was measured according to Holmes et al. (1998);
- Nitrate and phosphate content was determined as per standard method (APHA 1985);
- Total suspended solids were measured by oven-dried method (Wyckoff 1964);
- Fecal coliform (FC) following the Standard Procedures for Water Analysis sections 9221B and 9221E (APHA 1995).

Three parallel samples were taken from each measuring point for all parameters. The average values of the parallel measurements were used for the evaluation.

### Methodology

The protocol of the AEA method was devised by Németh et al. (2017). Five water quality classes and categories were used during the assessment of the aquatic environment index (AEI). The exclusive water source of the Lake is the River Nile inflow from the south, with a water yield of about 70 km^3^/year (Mageed and Heikal 2005). The legal limit values for the water parameters were determined from the pertaining specifications used for the quality categorization of the water parameters according to the Egyptian Governmental Decree No. 92/2013 (GD 2013). The weight indices (WI) of the water chemistry parameters were determined according to paired comparison (Németh et al. 2017).

## Results

The measured water chemistry parameters for the nine sampling sites around Lake Nasser and the limit values of Egyptian Governmental Decree No. 92/2013 (GD 2013) are summarized in Table 1.

Table 2 includes the legal limit values for the waters, and the ranking of water quality into five quality classes/categories according to the measured figures and specifications. The blue-highlighted boxes indicate the classes/categories in which the actual measured water chemistry parameters can be assigned. The ranking is illustrated based on measurement results of High Dam site as shown in Table 2.

**Table 2:** Measured values of the water chemistry parameters at Aswan High Dam no. 1 site. The italicized figures indicate the categories into which the measurement results fall according to Egyptian Governmental Decree No. 92/2013

Parameter	Measured value	Quality classes (QCi) and categories				
		I.	II.	III.	IV.	V.
		Bad	Weak	Proper	Good	Excellent
Chl-a (μg/L)	11.00	> 25	25	16.6	*13.3*	< 10.00
pH	7.66	> 11.00	11.00	10.00	9.00	*8.00–7.00*
NTU	8	> 50	50	25	*10*	5
EC (μs/cm)	250	> 1500	1500	1000	800	*< 600*
DO (mg/L)	4.50	< 4.00	*5.30*	6.66	7.51	> 10.00
COD (mg/L)	8.70	> 15.00	15.00	*10.00*	8.00	< 6.00
BOD_5_ (mg/L)	1.37	> 9.12	9.12	6.00	4.80	*> 3.60*
NH_4_ (μg/L)	10	< 500	500	330	260	*> 190*
NO_3_ (μg/L	2000	> 3000	3000	*2000*	1600	< 1200
NO_2_ (μg/L)	12	> 50	30	20	10	*< 10*
PO_4_ (μg/L)	220	> 750	750	500	400	*< 300*
TP (μg/L)	400	> 300	300	200	1600	*1200*
TSS (mg/L)	17	> 100	100	50	*20*	10
Fecal coliform (no./100 ml)	30	> 304	304	200	160	*> 120*

The chlorophyll-a value (11 μg/L) was categorized to quality class IV at High Dam, site no. 1, which is equivalent to quality category “good” since the measured concentration is below 13.3 μg/L according to Egyptian Governmental Decree No. 92/2013 (GD 2013). The pH was ranked to quality category “excellent” according to Egyptian Governmental Decree (GD 2013) regarding the measured value of 7.66.

The turbidity category was excellent with recorded value below 10 NTU. Nonetheless, it was good based on Egyptian Governmental Decree No. 92/2013. The DO category was weak because it was below 6 mg/L comparing with Egyptian Governmental Decree No. 92/2013.

**Fig. 2:**
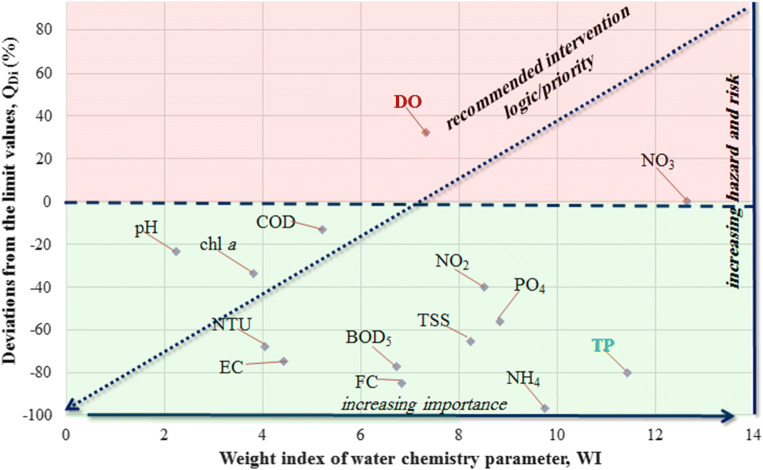
Deviation of water chemistry parameters from the limit values in function of the weight indices for Aswan High Dam site based on Egyptian Governmental Decree No. 92/2013

Total phosphorus value (0.40 mg/L) has been assigned into the excellent category as shown in Table 2. The ranking of the water chemistry parameters can be accomplished in the abovementioned way with using the interval confine values for the different categories. The individual “weights indices” (WI) for the water chemistry parameters are summarized in Table 3. If the water chemistry parameters show resembling results, then, environmental mitigation actions should be planned according to the weight indices (Fig. 2, *x* axis).

**Table 3:** The weight indices (WI) of the water chemistry parameters

Parameter	WI
Chl-a (μg/L)	3.82
pH	2.24
NTU	4.05
EC (μs/cm)	4.42
DO (mg/L)	7.32
COD (mg/L)	5.22
BOD_5_ (mg/L)	6.73
NH_4_ (μg/L)	9.75
NO_3_ (μg/L)	12.64
NO_2_ (μg/L)	8.51
PO_4_ (μg/L)	8.82
TP (μg/L)	11.42
TSS (mg/L)	8.23
Fecal coliform (no./100 ml)	6.83

By using Eq. (1), the distance/deviation values were determined for all 14 water chemistry parameters and for all measurement sites as given as follows:

1 $$ {\mathrm{Q}}_{\mathrm{Di}}=\frac{\left({\mathrm{C}}_{\mathrm{Mi}}-{\mathrm{C}}_{\mathrm{Lvi}}\right)}{{\mathrm{C}}_{\mathrm{Lvi}}}\times 100 $$

where QDi is the deviation of the measured water chemistry parameter i from the legal limit value for parameter i (%), C_Lvi_ is limit value of water chemistry parameter i, and CMi is the measured value of water chemistry parameter i.

The deviation of water chemistry parameter of the DO from the legal limit value can be given as follows:

$$ {\mathrm{Q}}_{\mathrm{DDO}\left(\mathrm{High}\ \mathrm{Dam}\right)}=\frac{\left(6.66-4.5\right)}{4.5}\times 100=32\% $$

The parametric level analysis of the water chemistry parameters is shown in Fig. 2. On the *y* axis (Fig. 2), the distance/deviation of the water chemistry parameter from the limit value (hereinafter referred to as distance/deviation) is plotted in the function of the weight indices.

If the deviation is positive like DO, it means that the water chemistry parameter considered does not meet the legal specification.

The aquatic environmental index (Table 4) is calculated according to Eq. (2).

**Table 4:** The AEI values for the measuring points around Lake Nasser

Number	Measuring point	Egyptian GD No. 92/2013	
		AEI value	Quality description
1.	Aswan High Dam	30.44	Good
2.	Dahmit	30.82	Good
3.	Kalabsha	29.38	Good
4.	Wadi-Abyad	30.15	Good
5.	El-Madiq	32.52	Excellent
6.	Korosko	31.09	Good
7.	Amada	31.98	Good
8.	Tushka	31.10	Good
9.	Abu-Simble	32.80	Excellent

2 $$ \mathrm{AEI}=\frac{\sum \limits_i^n={\mathrm{QC}}_{\mathrm{i}}\times {\mathrm{WI}}_{\mathrm{i}}}{n} $$

where AEI is the aquatic environment index; QC_i_ is the quality class for the water chemistry parameter i (on the basis of Table 2); WIi is the weight index for water chemistry parameter i (Table 3); *n* is the number of the water chemistry parameters (number of parameters used in the study).

The AEI values’ calculations are summarized in Table 4. Németh et al. (2017) summarizes the interpretation of the AEI values, and those are related to different water quality categories (excellent, good, proper, weak, and bad).

**Fig. 3:**
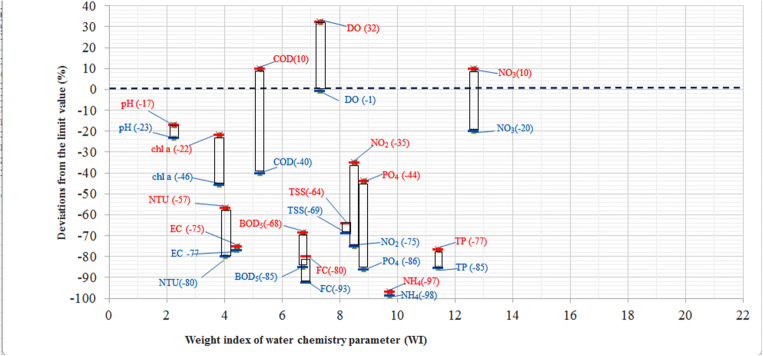
Deviations of the water chemistry parameters from the limit values, Q_Di_, based on Egyptian Governmental Decree No. 92/2013 in function of the weight index, where blue and red colors are best and worst values of the parameters, respectively

Substitution method was used for the determination of the mean AEI values and AEI intervals which are summarized in Table 5. The low and top limit values of the intervals were determined by the mathematical averaging of the neighboring AEI figures. For example, (7.14 + 14.29)/2 = 10.71. In this way, the top figure of the bad interval is equal to 10.71. Data summarized in Fig. 3 show the deviations of the water chemistry parameters from the limit values expressed in percentage for the nine measurement sites around the Lake Nasser.

**Table 5:** Evaluation categories for the quality classes

Category	Mean AEI value	AEI interval
Bad	7.14	10.71 ≤ AEI
Weak	14.29	10.71 ≤ AEI < 17.86
Proper	21.43	17.86 ≤ AEI < 25.00
Good	28.57	25.00 ≤ AEI < 32.14
Excellent	35.71	32.14 ≤ AEI

In Fig. 3, the highest distances/deviations are marked by red color and represent the most disadvantageous situation. It can be seen in Fig. 3 that the water quality is influenced mostly by dissolved oxygen, COD, and nitrate content. It can be seen that dissolved oxygen is far above the limit values in case of most measurement points, so it is defined as a highly important parameter due to the hot climate of Upper Egypt. The interpretation of the parameters is discussed in the literature (Németh et al. 2017).

It can be seen that the water quality of Lake Nasser is influenced mostly by dissolved oxygen content and nutrient concentrations according to the Egyptian Governmental Decree No. 92/2013 (GD 2013). The phosphorus content, nitrite, and nitrate content are very important due to their high eutrophication potential. For the achievement of significant improvement in the water quality of the Lake Nasser, the concentrations of nitrate intake should be decreased. From the point of view of the other water chemistry parameters, the water quality of the Lake can be qualified as “proper,” “good,” or “excellent.” The blue-colored-dashed line in Fig. 3 represents the concentration identical to the limit value. The points above the dashed line do not meet the environmental specifications. As seen in Fig. 3, the NO_3_ contents are above the limit values in case of two measurement points (Kalabsha and Tushka).

The water quality status exhibited “good” at seven sampling sites (High Dam, Dahmit, Kalabsha, Wadi-Abyad, Korosko, Amada, and Tushka) and exhibited “excellent” water quality at two sampling sites (El-Madiq and Abu-Simble regions). Lake Nasser maps were prepared on the basis of Google Maps available in Quantum GIS software to support the visualization of the AEI results. The measured values (in case of AEI, the calculated values) were placed onto the GIS layers of the Lake Nasser at nine measurement points.

**Fig. 4:**
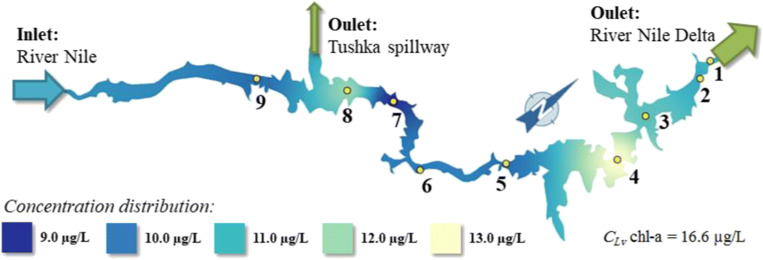
The chlorophyll-a concentration distribution in the Lake Nasser

The chlorophyll-a content of Lake Nasser is illustrated in Fig. 4. The chlorophyll-a concentration is changing between 9 and 13 μg/L, which are considered as “excellent” and “good” depending on the Egyptian GD No. 92/2013.

**Fig. 5:**
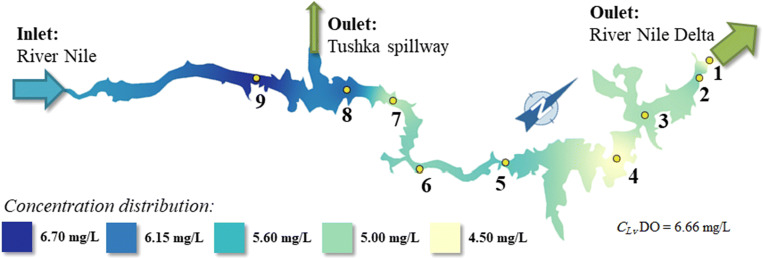
The dissolved oxygen concentration distribution in the Lake Nasser

The dissolved oxygen (DO) content is one of the most important water quality parameters; it has an important role for aquatic life and human consumption, and it is one of the limiting factors as defined in Egyptian Governmental Decree No. 92/2013 for water quality assessment. It was qualified as “good” at one site (Abu-Simble) and “proper” at two sites (El-Madiq and Tushka) and “weak” at six sites (High Dam, Dahmit, Kalabsha, Wadi-Abyad, Korosko, and Amada) in different sections of the Lake Nasser according to Egyptian Governmental Decree No. 92/2013 (Fig. 5).

**Fig. 6:**
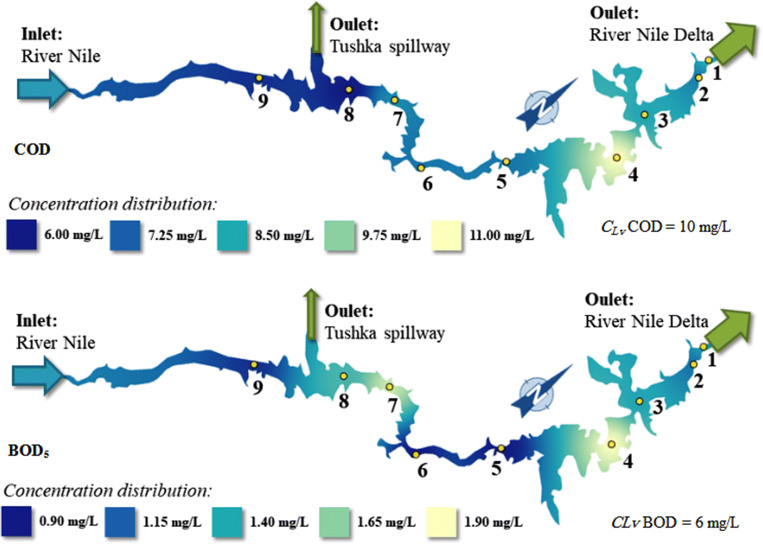
The chemical and biological oxygen demand concentration distribution in the Lake Nasser

The chemical oxygen demand varied between 6 and 11 mg/L. It can be qualified as “good” at six sites (Dahmit, El-Madiq, Korosko, Amada, Tushka, and Abu-Simble) and “proper” at two sites (High Dam and Kalabsha) and “weak” at one site (Wadi-Abyad). Figure 6 shows that a contaminated part of the Lake is at Wadi-Abyad site regarding the COD and BOD_5_ values due to the possible pollution effect of a nearby fertilizer-manufacturing factory which could result in some discharge into the Lake.

**Fig. 7:**
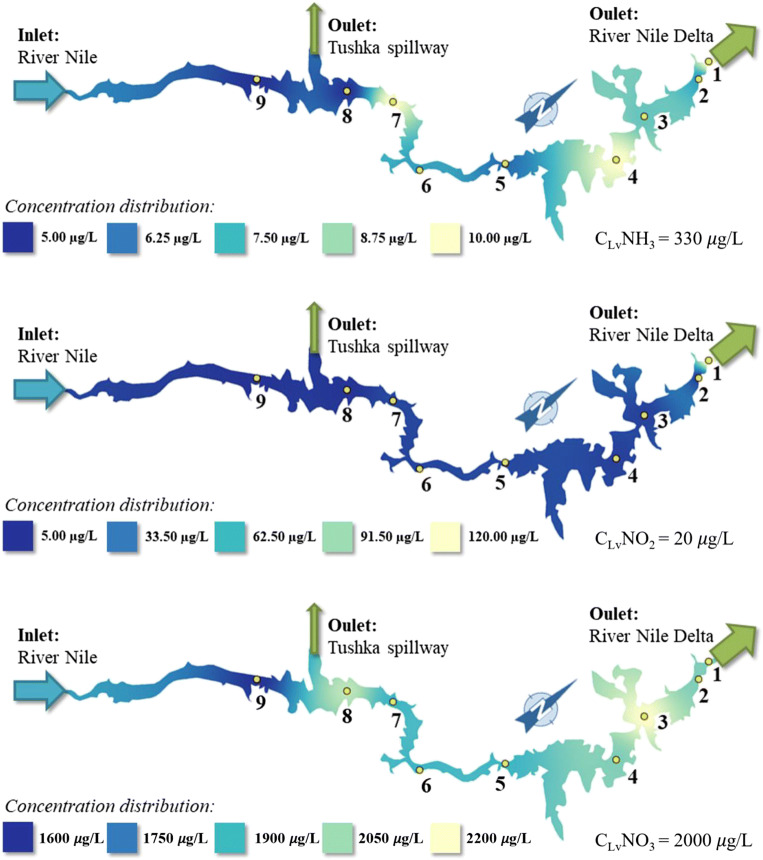
The N-nutrient concentration distribution in the Lake Nasser

Figure 7 shows the concentration distribution of N-nutrients along the Lake Nasser. The ammonium concentrations were below the limit value (0.33 mg/L) which can be categorized as “excellent.” The nitrite concentrations were approximately the same at all sites along the Lake. According to nitrate distribution, Kalabsha region showed the highest concentration at the Lake (2.2 mg/L), and at Tushka region, it was about the same (2.1 mg/L). These figures can be categorized as “weak”; however, those are still close to the limit value (2.0 mg/L).

**Fig. 8:**
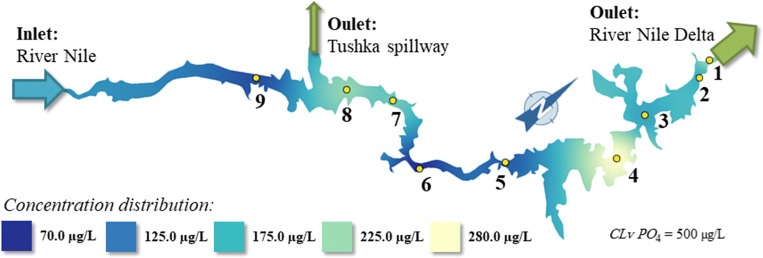
The orthophosphate concentration distribution in the Lake Nasser

**Fig. 9:**
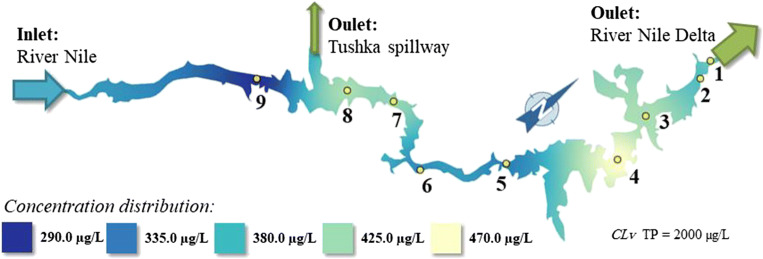
The total phosphate concentration distribution in the Lake Nasser

It can be stated that the blue-shaded areas in Figs. 8 and 9 represent concentration distribution of PO_4_-P and TP, respectively, which are below the permissible limit according to Egyptian GD No. 92/2013 (GD 2013).

**Fig. 10:**
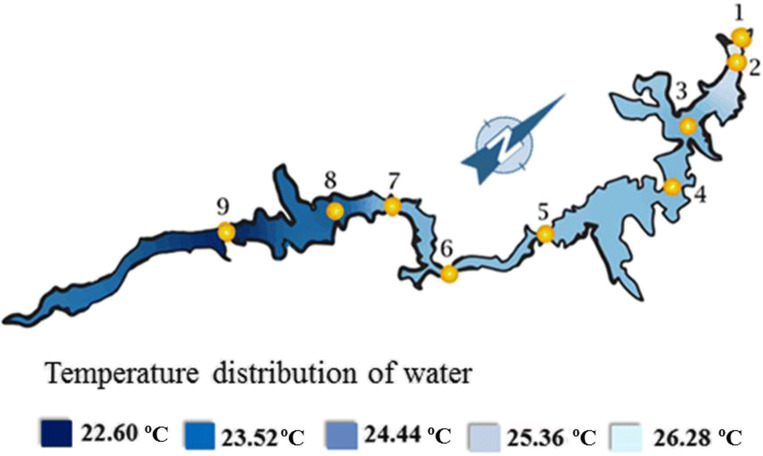
Water temperatures of the nine sampling sites around the Lake Nasser

**Fig. 11:**
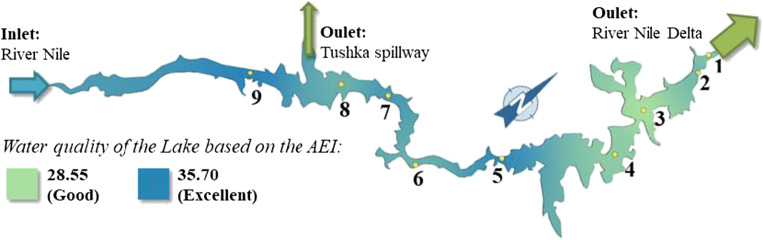
Water quality status among the basins of the Nasser Lake based on AEI values according to Egyptian Governmental Decree GD No. 92/2013

The AEI values (28.55 and 35.70) in Fig. 10 are the AEI results from the nine measurement sites as an outcome of the IDW-3D Analyst program used. The green-colored areas with AEI value of around 30.71 (the average of seven “good” sites) indicate the status of the water according to the specifications. The light green areas represent “good” water quality category, and the dark blue areas represent excellent water quality in Fig. 11.

## Discussion

There is a marked difference in the dissolved oxygen contents between the south and north parts of the Lake as shown in Fig. 5. This difference is may be due to the increase in water temperature (Fig. 10) (Matta et al. 2015; Song et al. 2019) and the decrease of wind speed at the Aswan High Dam site (Hussein and El Shafi 2005; El-Shazli et al. 2018).

The results showed that at the southern part of the Lake with average water temperature about 22.6 °C, the average dissolved oxygen content is 6.6 mg/L (Fig. 5). Moreover at the north part with average temperature of 26.3 °C, the average dissolved oxygen content is 5.13 mg/L, so the dissolved oxygen content is higher at the southern part of the Lake due to lower water temperatures (Fig. 10).

The dissolved oxygen content (DO) is of vital importance for most aquatic species. When the aquatic species are exposed to less than 2.0 mg/L oxygen for a short period of time, it may eliminate most of biota in the aquatic system (Geol et al. 1980 and Dodds 2006), while dissolved oxygen content values of 5.0 to 6.0 mg/L are usually fit for aquatic life (Singh et al. 2008 and Choi et al. 2017).

The chemical and biological oxygen demands provide information to calculate the organic matter content of the water (Mustapha et al. 2013; Ali et al. 2014; Bayard et al. 2018). The results indicated that COD and BOD_5_ concentrations were close to the limit values of clear waters. The contaminated part of the Lake which is represented by yellow color on the map (Fig. 6) is at Wadi-Abyad due to the runoff agricultural chemicals into the Lake. The increasing value of COD is in functional correlation with BOD. The increase in BOD concentrations at sites 4 and 8 is due to the organic matters stemming from the respiration of plankton and bacteria in the Lake (Osman et al. 2010 and Smagin et al. 2018).

Nitrogen containing nutrient concentrations in Lake Nasser is affected by a number of processes. Nitrate content is stemming from River Nile inflow from the south and nitrification of ammonia which is consumed by algal uptake during growth. The decrease in nitrate depends on the consumption of these ions by phytoplankton as well as their reduction by denitrifying bacteria (El-Otify et al. 2015).

This may be due to nitrification of NH_3_ and NO_2_ produced by the biochemical decomposition of descending dead plankton into nitrate by nitrifying bacteria. A slight decrease in the amount of nitrite especially in the surface water zone can be due to biological uptake in the photic zone (Soltan et al. 2005 and Painter et al. 2007). The absence of nitrite may be due to its oxidation process to nitrate as a result of well-oxygenated bight water. Sources of ammonia (NH_4_) include River Nile inflows from the south as shown in Fig. 7. The sinks include nitrification (conversion to nitrate), algal uptake during growth, and reservoir outflow (Cole and Wells 2008).

It can be supposed that the phosphorus load in the northern part of the Lake may stem from the discharge of agricultural effluents containing large amount of fertilizers (El-Shabrawy 2009) as indicated by light green–shaded areas in Figs. 8 and 9 where national agriculture projects in Wadi-Abyad and Tushka region (sites 4 and 8) are in operation. The average concentrations of total phosphorus ranged between 290 and 470 μg/L, and orthophosphate varied between 70 and 280 μg/L which is within the recommended limits of water quality in the River Nile (2 mg/L). Total and orthophosphate concentrations increased at sites 4 and 8 and those decreased downstream.

The agricultural runoffs (Goma 2000; El-Shinnawy 2002; Dumont 2009) into the Lake and pesticide residues from irrigated fields (Purandara et al. 2003; Abdelrazek 2019) result in phosphorus inputs into the water body of Lake Nasser. As a result of the changes in the biodiversity of Lake Nasser could occur (El-Shabrawy and Goher 2012; Salah El Din et al. 2015; El-Otify and Iskaros 2015). Phosphate concentrations in Lake Nasser increased southward and were weakly negatively correlated with phytoplankton biomass. Such negative relations were also found in some European temperate lakes (e.g., Napiórkowska-Krzebietke et al. 2013; Napiórkowska-Krzebietke and Hutorowicz 2015). Figure 10 shows that the water quality of the Lake Nasser on the basis of the aquatic environmental indices was determined. The results are in harmony with the report of the Ministry of Environmental Protection and Water Management of Egypt (AFDB 2015). Good water quality can be observed at the northern part of the Lake at site 3 which is indicated by light blue and green (Fig. 11).

Based on the outcome of the study, it can be stated that the AEA can be expediently used for the evaluation of the water quality changes and provides a collective indicator for mapping areas where the water quality is poor. In addition, AEA supports the prioritization of future mitigation actions.

The AEI map (Fig. 11) shows lower AEI values on the northern part of the Lake. This may be due to the use of fertilizers and pesticides in the agricultural areas and may suggest the intermittent supply of contaminating materials into the water body (Park 2001; El-Otify and Iskaros 2015).

## Conclusions and recommendations

Lake Nasser, a man-made Lake, represents the national freshwater bank of Egypt. It is recognized that improving the water quality management in Egypt is an indispensable mean of the future and a mean to cope with the challenges of water scarcity and healthy water supply. It has been defined as a crucial development objective in governmental plans. However, the increasing pressure on water resources due to the increasing demand for a rapidly growing population and the heavy financial burden to achieve this goal is a real challenge. An important factor affecting the AEI value in Lake Nasser is the water temperature. It is the most important factor affecting on most of the physical-chemical parameters in the water of Lake Nasser.

In conclusion, the status of water quality management can be summarized in terms of completed, realized, and ongoing procedures as follows: environmental protection procedures are being implemented, laws and regulations are in force, water quality standards are being developed, water monitoring and quality controls are in operation. Egypt has made remarkable progress in water quality management over the last decades. Water quality of Lake Nasser is good and safe for all uses.

## Nomenclature

AEA: Aquatic environmental assessment
AEI: Aquatic environmental index
APHA: American Public Health Association
BOD_5_: Biochemical oxygen demand during decomposition occurring over a 5-day period
Chl: Chlorophyll-a content
CLvi: Limit value of water chemistry parameter i
CMi: Measured value of water chemistry parameter i
COD_cr_: Chemical oxygen demand
DO: Dissolved oxygen content
EC: Electrical conductivity
GD: Governmental Decree
GIS: Geographic Information System
IDW: Inverse distance weighted
*n*: Number of the water chemistry parameters
NH_4_-N: Ammonium-nitrogen content
NO_3_-N: Nitrate-nitrogen content
PO_4_-P: Orthophosphate content
QCi: Quality class of water chemistry parameter i
QDi: Deviation of water chemistry parameter i from the legal limit value
TP: Total phosphorus content
NTU: Turbidity
WFD: Water Framework Directive
WI: Weight index
